# Influence of a Survival Swimming Training Programme on Water Safety Knowledge, Attitudes and Skills: A Randomized Controlled Trial among Young Adults in Sri Lanka

**DOI:** 10.3390/ijerph182111428

**Published:** 2021-10-30

**Authors:** Jeewanthika Ekanayaka, Chan Kim Geok, Bernadette Matthews, Samath D. Dharmaratne

**Affiliations:** 1 Department of Nursing, Faculty of Allied Health Sciences, University of Peradeniya, Peradeniya 20400, Sri Lanka; 2 Department of Nursing, Faculty of Medicine and Health Sciences, Universiti Malaysia Sarawak, Kota Samarahan 94300, Sarawak, Malaysia; kgchan@unimas.my; 3 Research and Health Promotion Department, Life Saving Victoria, 200 The Boulevard, Port Melbourne, VIC 3207, Australia; bernadette.matthews@lsv.com.au; 4 Department of Community Medicine, Faculty of Medicine, University of Peradeniya, Peradeniya 20400, Sri Lanka; samath.dharmaratne@mbbs.md; 5 Department of Health Metrics Sciences, Institute for Health Metrics and Evaluation, School of Medicine, University of Washington, Seattle, WA 98195, USA

**Keywords:** drowning, water safety knowledge, water safety attitudes, survival swimming skills, water safety education, young adults

## Abstract

Drowning among young adults is high in Sri Lanka. Water safety education is a recommended strategy for drowning prevention but is often overlooked for young adults. This study aimed to evaluate the effectiveness of an adapted educational intervention, “Swim for Safety” on improving water safety knowledge, attitudes and survival swimming skills among undergraduates (19–28 years) in Sri Lanka. This study employed a parallel-group, two-arm randomized controlled trial design. The intervention group (*n* = 78) received a face-to-face, 12-lesson education programme, and the control group (*n* = 78) received a brochure and weekly mobile phone messages for six consecutive weeks. Baseline, post-intervention and three-month follow-up knowledge, attitudes and skills were evaluated. Knowledge and attitudes were assessed using a self-administered questionnaire and skills were evaluated following a skills assessment protocol. In total 116 participants, 60 intervention group and 56 control group, completed the study. At baseline there were no differences between groups in median scores of water safety knowledge, attitudes and survival swimming skills. The intervention group demonstrated statistically significant increases in median water safety knowledge, attitudes and survival swimming skill scores compared with the control group, following the intervention and maintained at three-month follow-up (*p* < 0.05). The adapted Swim for Safety programme significantly improved water safety knowledge, attitudes, and survival swimming skills among young adults in Sri Lanka. Therefore, it is recommended that the SfS programme be implemented widely to prevent drowning in young adults.

## 1. Introduction

Drowning is a major cause of unintentional injury and a public and global health issue that leads to death and disability [1,2]. An estimated 320,000 people die from drowning annually worldwide, with low and middle-income countries accounting for over 90% of unintentional drowning deaths [2]. However, these figures are underestimated as they do not include deaths caused by flood disasters or water transport incidents [3,4]. Both fatal and non-fatal injuries related to drowning account for high healthcare costs and negative psycho-social and economic impacts on the victim, family, and society [2,3,4,5,6]. In many countries, young adults (18–34 years) are a high-risk group for drowning. Particularly those under 25 years, accounting for 50% of global drowning-related deaths [2]. Prevention strategies typically focus on children (<18 years) [7,8].

In Sri Lanka, the death rate due to drowning is 3.5 per 100,000 population, ranking 12th compared to 35 other low and middle-income countries [9]. The age-specific crude drowning rate increased significantly from age 15–24 years with 4.7 per 100,000 population per annum [9]. The young adult population, aged between 15–24 years, accounts for approximately 20% of drowning deaths [9]. Aquatic risk-taking behaviors including consuming alcohol in, on, or around water are reported to contribute to drowning among young adults [10,11,12]. Literature indicates that seeking increased independence, peer influence and shaping and forming an identity among peers are significant influences on young adult risk-taking behaviors [13]. Furthermore, the lack of water safety knowledge, attitudes, and survival swimming skills are also key factors for unintentional drowning [14,15]. Therefore, educating young adults on water safety that aims to improve water safety knowledge, attitudes, and survival swimming skills is vital to prevent unintentional drowning in this population [15,16].

University students (19–28 years) are one of the young adult groups at high-risk of drowning [17]. The National Action Plan for Drowning Prevention and Water Safety in Sri Lanka highlights the need for drowning prevention among university students in Sri Lanka [18]. However, both locally and worldwide, most water safety training programmes and research studies have targeted children [19,20,21,22,23], while the young adult population seems to have been neglected [16]. According to the author’s knowledge, the available water safety training programmes and research studies for university students or young adults have been conducted in high-income countries which have a different pattern of drowning compared to Sri Lanka [16,17,24]. Scientifically proven or validated water safety training programmes targeting the improvement of survival swimming skills for university students or young adults have not been found in low-and-middle-income countries. The effectiveness of drowning prevention programmes depends on the age, socio-demographic and cultural background of the target population [16,25]. Therefore, training programmes developed for other countries or other age groups may be inapplicable to young adults/undergraduates in Sri Lanka.

To the authors’ knowledge, there are no published studies that examined the influence of water safety education on water safety knowledge, attitudes and skills among university students in Sri Lanka. Therefore, this study aimed to evaluate the influence of an adapted educational programme, Swim for Safety (SfS) on water safety knowledge, attitudes, and survival swimming skills among undergraduates in a public university in Sri Lanka.

## 2. Materials and Methods

### 2.1. Study Design

This study employed a parallel-group, two-arm, randomized controlled trial, which consists of intervention and control groups that involved a pre-test post-test, follow-up design. The SfS programme was delivered to the intervention group, while printed brochures and weekly mobile phone messages on drowning prevention and water safety awareness were given to the control group.

### 2.2. Study Setting

This study was conducted at the University of Peradeniya, Sri Lanka. It is the largest university in Sri Lanka, offering a variety of studies including medicine, science, humanities, and engineering. It is located in the Central Province, approximately 8 km from the major city of Kandy.

### 2.3. Study Population

The population of the study was all 2872 first-year students in the university. According to the student registration division of the University of Peradeniya, the student population consisted of 38.5% male and 61.5% female undergraduates. More than 80% are Sinhalese with varied socio-economic backgrounds. All first-year undergraduates in each faculty are considered to have similar characteristics as they enrolled in university just after completing their school life around 19 years and belong to the same age group. Furthermore, as there was no exposure to survival swimming and water safety education in the Sri Lankan school system, all students were likely to have a similar level of water safety knowledge, attitudes, and survival swimming skills.

### 2.4. Study Participants

This study was open to all first-year undergraduate students from all non-health related faculties of the University of Peradeniya, Sri Lanka. Exclusion criteria were students who: (1) had any previous formal swimming, lifesaving, or Basic Life Support (BLS) training; (2) self-reported any physical or mental disorders (e.g., epilepsy, physical disabilities) that can increase drowning risk; (3) were unable to speak Sinhala as the medium of instruction in the SfS programme was Sinhala language, and it is one of the main languages spoken in Sri Lanka; (4) were from a health-related faculty because of assumed prior knowledge of some components of the intervention (e.g., pathophysiology of drowning, Basic Life Support) during their university education).

### 2.5. Sample Size

The minimum total sample size was calculated using G* Power software (version 3.1.9.7) with, (a) significance level (α): 0.05 [26], (b) effect size 0.6 (c) power 0.95 (d) non-response rate: 26.4% [19]. The effect size and non-response rate were obtained by calculating the unpublished first-year data from the SfS programme for school children in Sri Lanka, with the permission of Life Saving Victoria (LSV) and Sri Lanka Life Saving (SLLS). The sample size calculated was 122 plus 34 (estimated for dropout participants), i.e., a total of 156 participants with 78 allocated for the intervention group and 78 for the control group.

**Figure 1:**
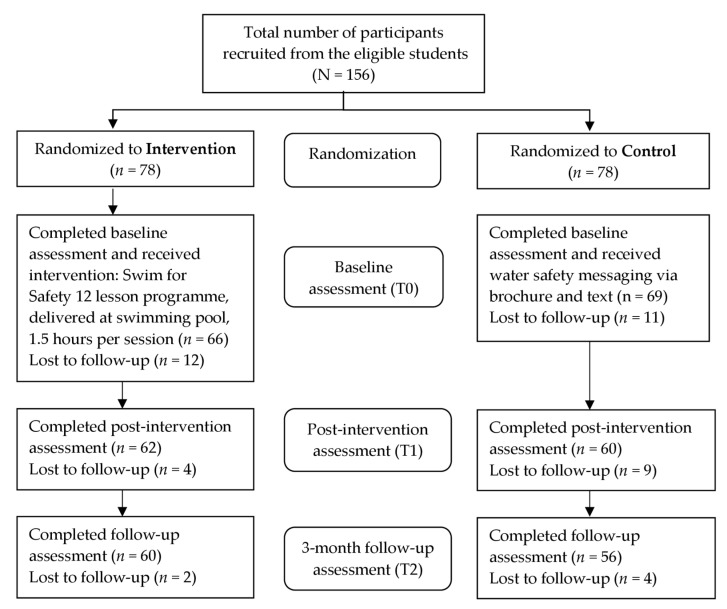
Flow diagram of participants through the trial and data collection. Water safety knowledge, water safety attitudes and survival swimming skills were assessed at the baseline (T0), immediate post intervention (T1), and three-month follow-up (T2).

In total 116 participants completed the study (Figure 1), including 60 in the intervention group and 56 in the control group with response rates of 76.9% and 71.8%, respectively. Reasons for drop out included: participants’ decision not to participate in the study following the initial provision of consent; missing one or more lessons in the intervention group; leaving the study during the intervention, and absence at the time of post-intervention and/or follow-up assessments.

### 2.6. Recruitment

All first-year students were invited to the study after properly explaining the purpose, risk and benefits of the study, safety measures employed during the programme and role of the participants and inclusion and exclusion criteria of the study. A list of the students who were eligible to participate in the study was obtained. Simple random sampling was used. A total of 156 participants were randomly assigned to either intervention (*n* = 78) or control (*n* = 78) group using a list of computer-generated random numbers. 

### 2.7. Intervention

The original SfS programme was first developed and implemented in Australia by LSV [27]. This programme has been validated and culturally adapted for school children in Sri Lanka by LSV with SLLS and is ongoing [19,28]. The present study used an adapted version of SfS programme from the ongoing nationwide SfS programme for school children in Sri Lanka [29]. The adaptation process was informed by a literature review of other relevant water safety programmes and guidelines [16,24,30,31,32,33,34,35] and finalized based on a review by ten survival swimming and water safety experts and a pilot study with 26 undergraduate students.

The intervention group received the adapted SfS programme for undergraduates, which consisted of 12 face-to-face programme lessons. The programme lessons were conducted in the university swimming pool. The duration per lesson was 90 min, delivered two days per week, over six consecutive weeks (a total of 18 h of instruction per group). The language medium of instruction for the training was Sinhala. The components of the SfS programme are listed in the following Box 1.

Box 1 **Overview of programme components.**

- Importance of water safety education and survival swimming
- Hazard identification in, on and around different aquatic environments
- Common high-risk aquatic activities and factors that lead to drowning
- Safety signs
- Basic boating safety
- Safe rescue
- Safe entries (slide in and compact jump) and exits
- Breath control
- Movements in and through water
- Sculling and treading water
- Floating
- Rotation
- Swimming: survival backstroke, survival breaststroke
- Basic Life Support following drowning (DRSABCD)
- Role play on Emergency Scenario to cover all the knowledge and skills

Participants in the control group were each given a brochure on drowning prevention and water safety. The brochure consisted of standard information on drowning prevention and water safety used by SLLS and the World Health Organization (WHO) [9,30]. The information included: current drowning trends, causes of drowning, how to identify a drowning victim, the role of young adults in drowning prevention, and ten recommendations for drowning prevention. It was delivered to the participants in the control group through appointed research assistants from each faculty. Furthermore, a weekly mobile phone message with brief water safety messaging together with a reminder to read the brochure was sent to the participants by the researcher (Box 2).

Box 2 **Example text messages sent to control group.**

1. Drowning is a major type of unintentional injury. Let’s be safe around the open water bodies.
2. Accidental drowning kills >855 people in Sri Lanka each year. Let’s avoid risky aquatic activities.
3. Drowning can be prevented. Unsafe and unskilled rescue leads to multiple drownings.
4. Males are four times more likely to drown than females. Let’s be safe around the water.
5. Being in the water is fun. But unsafe activities can kill you. Let’s learn to be safe on, in and around water.

Absentees for any lesson of the intervention or assessment were considered dropouts to avoid potential bias from missed lessons. They were still allowed to participate however their results were not included in the final analysis. The programme was conducted by a trained team from SLLS that consisted of a head coach, an assistant coach, six lifesavers, and four assessors, who had previously undertaken the formal SfS training with SLLS and LSV.

### 2.8. Preventing Contamination

The risk of possible contamination was avoided by educating the participants not to share the information they learned during the training with other students who were not in the intervention group and thoroughly explaining the effects of contamination on the study and possible disadvantages [36,37].

### 2.9. Data Collection

Participants’ water safety knowledge, attitudes, and survival swimming skills were assessed before the intervention i.e., baseline (T0), immediately after the completion of 12-lesson training programme i.e., immediate post-intervention (T1), and at three months after the intervention i.e., three-month follow-up (T2) (see Figure 1).

#### 2.9.1. Knowledge and Attitudes Assessment

Water safety knowledge and attitudes were assessed using an adapted and validated Sinhala version of the self-administered questionnaires. The self-administered questionnaire consisted of two parts, part A to assess socio-demographic characteristics and background information on previous drowning, rescue and aquatic experiences and part B to assess water safety knowledge and attitudes. Only part B was administered at immediate post-intervention and three-month follow-up. The questionnaire was administered at the swimming pool premises and took approximately 15 min to complete. The completed questionnaires were collected straight after completion at the location to avoid any missing data.

#### 2.9.2. Skills Assessment

The skills assessment was conducted at the university swimming pool. Nine survival swimming skills including pull rescue, throw rescue, safe entry, safe exit, floating up to a maximum two minutes, rotation, BLS, reading safety signs (prohibition sign, warning signs) and flags (swim between flags and do not swim flag) and swimming up to 50 m were assessed. The assessment was conducted by two panels of trained assessors, which consisted of two assessors for each panel to avoid subjectivity of the findings [38,39]. Assessors were provided a skills assessment protocol to follow and data collection sheet to mark the allocated score for each skill.

### 2.10. Data Collection Instruments

#### 2.10.1. Knowledge and Attitudes Questionnaire

The questionnaire to assess water safety knowledge and attitudes was developed using questions adapted from the SfS knowledge and attitude questionnaire for school children in Sri Lanka, the literature [16,17,19,30,33,40] and expert comments based on the adapted SfS curriculum. Table 1 provides examples of the statements used. The knowledge section consisted of 15 questions. Participants responded using ‘yes’, ‘no’, and ‘do not know’ options. Marks were allocated for each knowledge statement with one mark for a ‘correct’ answer and zero marks for ‘incorrect’ or ‘do not know’ answers. Thus, the maximum possible score for the knowledge section was 15, while the minimum possible score was zero.

The attitude section consisted of 17 positive and negative statements. Participants responded using a five-point Likert scale (1-strongly agree, 2-Agree, 3-Neutral, 4-Disagree, 5-Strongly disagree) for each statement. Marks were allocated based on the level of agreement for each statement. The negative statements which were responded with ‘strongly disagree’ were allocated with a score of 5, while ‘strongly agree’ was allocated with a score of 1. The positive statements were given a reversed score. Thus, the maximal possible total attitudes score was 85, and the minimum possible score was 17.

The internal reliability of the 15-item water safety knowledge and the 17-item water safety attitude scales of the questionnaire used were analyzed. The Cronbach’s alpha for knowledge scale was α > 0.6 (0.66), which was considered acceptable (0.7–0.6) and for the attitude scale, α > 0.7 (0.72), which was considered good (0.8–0.7) [41].

#### 2.10.2. Skills Assessment Protocol

The skills assessment protocol was adapted from the SfS programme for school children [19,29] and expert comments. The protocol included how the assessment should be performed, how to arrange the assessment setting (swimming pool), the equipment to be prepared beforehand, how to deliver the instructions to the participants and how to score the performance of each skill. The assessors used the data collection sheet to enter the scores for each skill. There was a set of steps to be completed by the participants relevant to each skill. Assessors provided a score based on the completed step/s within each skill. For the skills such as, throw rescue, and safe entry one mark was provided if the participants completed the task successfully and zero if it was not completed successfully or not attempted. Floating was scored 0–2 marks, 0 if not completed, 1 if completed up to 1 min and 2 marks if completed 2 min. BLS was scored from 0–6 based on the level of completion of each step of DRSABCD with 0 if not attempted or incorrectly performed. Swimming distance was scored on a scale from 1 to 5 if completed 1–12 m, 13–25 m, 26–37 m, 38–49 m, and >50 m respectively and 0 if the task was not attempted. For swimming distance the technique/style of swimming was not scored. The maximum possible total skills score was 22, and the minimum possible score was zero.

### 2.11. Data Analysis

Data were analyzed using Statistical Package for the Social Sciences (Version 22). Descriptive statistics (frequencies, percentages, means) were used to characterize the sample. Statistically significant differences in demographic characteristics between the intervention and control groups were assessed using the Chi-square test for homogeneity or Fishers’ Exact test based on the normality of the data.

The percentages of total water safety knowledge, water safety attitudes, and survival swimming skills scores were categorized based on Bloom’s cut-off points, low (<60%), moderate (60–79%), and good (80–100%) [42].

Data distribution was not normal on the Kolmogorov-Smirnov test. Non-parametric tests were used to compare total water safety knowledge, water safety attitudes, and survival swimming skills scores over three different time points and between groups. Wilcoxon signed-rank test was conducted to determine the effect of the SfS programme on water safety knowledge, attitudes, and survival swimming skills over three-time points, baseline, post-intervention, and follow-up, with a *p*-value significance level ≤ 0.05. Mann-Whitney U test was conducted to compare intervention and control groups to determine the effect of the SfS programme on water safety knowledge, attitudes, and survival swimming skills between groups, with a *p*-value, significance level ≤ 0.05.

### 2.12. Safety of the Participants during the Training and Assessment

To ensure the safety of participants during testing and the training programme, six lifesavers were allocated during each session. Non-swimmers (students who have never swum before) were not allowed into the pool during the pre-intervention assessment of both groups and post-intervention and follow-up assessments of control groups to avoid unintentional drowning. The participants were positioned and directed in the water to facilitate direct observation by the coaches and assessors.

## 3. Results

### 3.1. Socio-Demographic Characteristics of the Participants

The majority of participants in both groups were female, aged 21–24 years. Table 2 shows the socio-demographic characteristics of the participants. There were no statistically significant differences in demographic characteristics between intervention and control groups except their faculty of study. 

### 3.2. Levels of Knowledge, Attitudes and Survival Swimming Skills

Table 3 compares the change in the levels of water safety knowledge of intervention and control groups at each time point which has been obtained based on Bloom’s cut-off points [42].

Nearly all participants in both groups had a poor level of water safety knowledge at baseline. However, in the post-intervention and at follow-up, none of the intervention group had a poor level of knowledge. In contrast, more than 80% of participants in the control group remained at a poor level of knowledge in the post-intervention and follow-up. More than 90% of participants in the intervention group obtained a good level of knowledge post-intervention and follow-up, while none of the control participants achieved a good level of knowledge either post-intervention or at follow-up.

At baseline, more than 50% of participants in both groups had moderate attitude levels, and nearly 40% of participants in both groups had good attitude levels. At the post-intervention and follow-up, all participants in the intervention group and more than 60% in the control group achieved a good attitude level.

All participants in both groups had a poor level of survival swimming skills at baseline. More than 90% of participants in the intervention group increased their survival swimming skills to either a moderate or good level post-intervention and follow-up. In contrast, all the participants in the control group remained at a low level of skills at all three time points of assessment.

### 3.3. Comparison of Water Safety Knowledge Scores within and between Groups

Table 4 compares the median water safety knowledge, attitudes and survival swimming skills scores within groups and Table 5 compares those between groups.

There were no differences between intervention and control group median scores at baseline (T0). Immediately following the intervention, both groups had statistically significantly increased knowledge scores (Mdn Diff 7 intervention vs. Mdn Diff 1 control group). Both groups also had statistically significantly increased attitude scores (Mdn Diff 13.5 intervention vs. Mdn Diff 3.5 control group). But only the intervention group had statistically significant improvement in survival swimming skills (Mdn Diff 17 intervention vs. Mdn Diff −1 control). From immediate post-intervention to three-month follow-up, there were no significant median score changes in knowledge, attitudes and skills of either group (*p* > 0.05) (Table 4).

The median scores of water safety knowledge, attitudes and survival swimming skills were not statistically significantly different within intervention and control groups at baseline (*p* > 0.05). However, there were statistically significantly higher median scores in the intervention group at immediate post-intervention versus the control group (*p* > 0.05). This difference was maintained at three-month follow-up (Table 5).

## 4. Discussion

This study, conducted with the aim of evaluating the effectiveness of the adapted water safety training programme, namely “Swim for Safety,” demonstrated improvements in knowledge, attitudes, and skills among first-year undergraduates in Sri Lanka.

Evidence, including that from drowning reports and recent news reports, supports that young adults including undergraduates in Sri Lanka are at risk of drowning [9]. Key factors in drowning have been linked to issues arising from a lack of awareness and understanding of water dangers, increased aquatic risk-taking behaviors, and poor swimming skills [30]. Water safety education and survival swimming programmes are recommended for providing water safety knowledge and skills, improving water safety attitudes and promoting safe behaviors in and around different aquatic environments [43].

Randomized control trials are the gold standard to assess the effectiveness of an educational intervention [44]. However, most research in this area included one sample pre- and post-interventional studies [16,19,21,45]. In the Sri Lankan context, randomized control trials targeting water safety education programmes have not been cited. This highlights the importance of the current study by providing evidence for a water safety-related education programme.

The current study found that the majority of the participants in both intervention and control groups had poor water safety knowledge at baseline. Post-intervention water safety knowledge of the intervention group was significantly improved compared to baseline with more than 90% of participants in the intervention group obtaining a good level of knowledge post-intervention and follow-up, while none of the control participants achieved a good level of knowledge either post-intervention or at follow-up. Whilst the control group showed statistically significant improvement in median knowledge score this was minimal (Mdn Diff 1) compared to the intervention group (Mdn Diff 7). The slight increase in knowledge score of the control group may be due to the drowning prevention and water safety awareness messaging in the brochure and text messages. Previous studies in other age groups and countries have similarly shown increases in water safety knowledge delivered via various methods [16,19,46].

The majority of participants in both groups had a moderate level of positive attitudes towards water safety at baseline. Post-intervention attitudes were significantly improved in the intervention group with 100% achieving a good attitude level compared to baseline. The improvement was retained with no significant change at three-month follow-up. In contrast, 69.6% of the control group had improved water safety attitudes at post-intervention and 60.9% at three-month follow-up. Similar to the knowledge score the increase in water safety attitudes of the control group may have been due to the water safety messaging provided in the brochure and text messages.

The baseline attitude level of both groups was higher compared to their baseline knowledge and skills. However, according to the author’s observations, the behaviors of the participants at the swimming pool at the beginning of the SfS programme showed poor attitudes on survival swimming and water safety. This may indicate a social desirability bias in the responses, whereby the participants tended to select the answers based on their perception of the appropriate attitude statement instead of providing a true response as to their actual attitudes. In any case there were still significant between-group differences in levels of improvement in water safety attitudes in the intervention group compared to the control group, demonstrating the benefits of the SfS programme for improving attitudes. As noted above, previous studies have reported improvements in water safety attitudes, however, these have focused on younger children or those with higher baseline levels of aquatic exposure and also did not use a control group [16,19,46].

The survival swimming skills (including, rescue, safe entry and exit, floating, rotation, BLS, reading safety signs and swimming competency) among the participants of both groups were poor at baseline. Post-intervention the skills of participants in the intervention group (Mdn (IQR) 18 (16–20.75) out of 22) were significantly improved compared to baseline (Mdn (IQR) 1 (0–2)) and were retained at three-month follow-up. Survival skills in the control group remained poor without any significant improvement over time. The findings demonstrate the effectiveness of the SfS programme for developing survival swimming skills for undergraduate students.

A previous study evaluated the effect of a 12-week short water safety intervention on water safety knowledge, attitudes, and skills among 154 exercise and sports science students in Australia [16]. The one-sample pre-post intervention study assessed water safety knowledge, attitudes, and skills at two-time points, at baseline and immediate post-intervention. The education programme was found to have improved water safety knowledge, attitudes and skills. However, given the students were exercise and sports science students they would be expected to be more advanced and with increased aquatic exposure which limits the generalizability of findings. The students in the current study were from a variety of faculties and had poor baseline skill levels and water safety knowledge. This therefore indicates that the results can be applied to the general young adult population.

Within-group comparisons showed that both knowledge and attitudes were increased in both the intervention and control groups following educational events. However, the SfS educational programme significantly improved the survival swimming skills of its participants and was more effective in increasing the level of knowledge and attitudes than providing a brochure and preventive messages alone. This suggests that even though knowledge and attitudes can be improved to some extent by providing standard information through print and other media, crucial survival swimming skills development require practical education such as that provided through the SfS programme.

Previous studies highlight that the learned content, especially skills may not be retained if participants do not practice or use them often [16,47]. Therefore, this study also included a three-month follow-up as a measure of short-term retention. The study findings demonstrated that the improvements in knowledge, attitudes, and skills obtained through the SfS programme were retained for at least three months without any significant reduction. However, the exact duration retention over a longer-period is uncertain.

Retention of knowledge, attitudes and skills can be influenced by the frequency of utilization after training [16,47]. The current study did not assess whether the retention of knowledge, attitudes and skills was influenced by the frequency of utilizing the learned content after the programme or any other water safety education they may have received after SfS. Given there are limited programmes available for young adults it is unlikely that they would have had the opportunity for continued practice. However, this is a limitation of the study. Further research studies are suggested to determine the retention of water safety knowledge, attitudes, and skills over time and determine the ideal length of time between training programmes and subsequent refresher programmes.

Previous studies have noted the importance of teaching survival swimming skills under conditions similar to real-life situations, such as performing skills in waves, rough water and clothes [48,49]. However, the current study focused on assessing the skills at the pool under controlled conditions. The SfS programme addressed this to a degree by providing participants with real-life scenarios and rough water through play activities and role play. Even though the study was initially planned to have a lesson component to practice skills with clothes on, the content was removed due to rules and regulations of the university swimming pool which did not allow entry in the pool with regular clothing. Further studies are recommended to include more real-life situations, in waves and with clothes, where possible and whilst also retaining safety, ensuring that participants are at an appropriate skill level to incorporate such activities.

## 5. Conclusions

Overall, the SfS programme was demonstrated to be effective in improving the water safety knowledge, water safety attitudes, and survival swimming skills of participants. Therefore, it is recommended that such programmes are delivered more widely to young adults. More specifically, the SfS programme is recommended for Sri Lankan universities to deliver the programme as one of the routine programmes offered to students. Further, the trained students from the study could be trained to provide public awareness programmes to improve knowledge and attitudes or undertake more advanced survival swimming and lifesaving courses and deliver future programmes.

Drowning is a public health problem of increasing concern, and its prevention is a multidisciplinary task. Public healthcare professionals all have a role to play in partnership to prevent unintentional drowning together with experts in water safety, including local lifesaving associations. Therefore, it is important to strengthen these partnerships for the prevention of drowning in Sri Lanka.

## Figures and Tables

**Table 1:** Example knowledge and attitude statements.

Statements
**Knowledge**
Good swimmers can swim alone, while non-swimmers should be accompanied a buddy.
2. At the beach, swim only in the area between the red flags.
3. Alcohol is a risk factor for drowning
4. Putting feet first is the safest way of entry to the water
5. Check risky conditions before entering the water.
6. A person should panic if they get into danger in the water.
7. If someone is drowning, a swimmer should jump into the water to save them.
8. If someone is drowning, the emergency phone number to call is 1919
**Attitudes** If someone I know is drowning, I will jump into the water to rescue him/her without thinking about myself. If someone is confident about their swimming ability, that person does not need to wear a lifejacket while boating. It is alright for good swimmers to swim/bathe in places mentioned as dangerous. If there are no lifeguards/police around, I would get into the water that mentioned not to swim. Swimming alone is not risky for me if I am a good swimmer. It is ok for someone to jump from high to dive in unfamiliar water bodies if he/she knows how to perform a safe dive. Someone does not need to learn survival swimming and water safety skills if he/she is not going to bathe/ swim in open water bodies (beach/river/lake/pool etc.).

**Table 2:** Socio-demographic characteristics of participants (N = 116).

Characteristic	Intervention Group (*n* = 60) *n* (%)	Control Group (*n* = 56) *n* (%)	Test Value	Df	*p* Value
Age					
Mean (SD)	22.0 (±0.759)	22.02 (±0.924)	−0.113	114	0.911 ^a^
21 years	6 (26.7%)	19 (33.9%)			
22 years	29 (48.3%)	22 (39.3%)			
23 years	14 (23.3%)	10 (17.9%)			
24 years	1 (1.7%)	5 (8.9%)			
Sex					
Male	15 (25.0%)	20 (35.7%)	1.578	1	0.209 ^b^
Female	45 (75.0%)	36 (64.3%)			
Faculty					
Agriculture	14 (23.3%)	9 (16.1%)	27.387	3	0.000 *^,b^
Arts	24 (40.0%)	10 (17.9%)			
Science	22 (36.7%)	17 (30.4%)			
Management	0 (0%)	20 (35.7%)			
Nationality					
Sinhala	60 (100%)	56 (100%)			Constant
Religion					
Buddhist	55 (91.7%)	53 (94.6%)			0.718 ^c^
Catholic	5 (8.3%)	3 (5.4%)			
Ever been in deep water (above waist height)					
Yes	59 (98.3%)	54 (96.4%)			0.737 ^c^
No	1 (1.7%)	2 (3.6%)			
Ever tried swimming					
Yes	56 (93.3%)	51 (91.1%)			0.737 ^c^
No	4 (6.7%)	5 (8.9%)			
Can swim (perform any propulsive movement of the body through the water using hands and legs, regardless of the technique)					
Yes	36 (60.0%)	23 (41.1%)	4.152	1	0.063 ^b^
No	24 (40.0%)	33 (58.9%)			
Total	60 (100%)	56 (100%)			

^a^ = *t*-test, ^b^ = Chi-square test, ^c^ = Fisher’s exact test, Df = degree of freedom, * significant difference between groups.

**Table 3:** Levels of water safety knowledge, attitudes and survival swimming skills—intervention vs. control group at each time point of assessment (N = 116).

Outcome	Level *	Intervention Group (*n* = 60)			Control Group (*n* = 56)		
		T0 *n* (%)	T1 *n* (%)	T2 *n* (%)	T0 *n* (%)	T1 *n* (%)	T2 *n* (%)
Knowledge	Poor	57 (95.5)	0 (0.0)	0 (0.0)	54 (96.4)	47 (83.9)	50 (89.3)
	Moderate	3 (5.0)	8.3 (5)	6 (10.0)	2 (3.6)	9 (16.1)	6 (10.7)
	Good	0 (0.0)	55 (91.7)	54 (90.0)	0 (0.0)	0 (0.0)	0 (0.0)
	Poor	0 (0.0)	0 (0.0)	0 (0.0)	1 (1.8)	0 (0.0)	0 (0.0)
Attitudes	Moderate	34 (56.7)	0 (0.0)	0 (0.0)	34 (60.7)	17 (30.4)	22 (39.3)
	Good	26 (43.3)	60 (100)	60 (100)	21 (37.5)	39 (69.6)	34 (60.7)
Skills	Poor	60 (100)	6 (10.0)	4 (6.6)	56 (100)	56 (100)	56 (100)
	Moderate	0 (0.0)	21 (35.0)	28 (46.7)	0 (0.0)	0 (0.0)	0 (0.0)
	Good	0 (0.0)	33 (55.0)	28 (46.7)	0 (0.0)	0 (0.0)	0 (0.0)

* Poor = <60%, Moderate = 60–79%, Good = 80–100%, T0—Baseline, T1—Immediate post-intervention, T2—three-month follow-up.

**Table 4:** Comparison of change in median water safety knowledge, attitudes and survival swimming scores within groups (N = 116).

Outcome	Time Points	Intervention Group			Control Group		
		Mdn Diff	Z	*p*-Value	Mdn Diff	Z	*p*-Value
Knowledge	T0 vs. T1	7	6.751	0.000 *	1	3.045	0.002 *
	T1 vs. T2	0	0.590	0.555	−1	1.192	0.233
Attitudes	T0 vs. T1	13.5	6.742	0.000 *	3.5	5.887	0.000 *
	T1 vs. T2	−0.5	−1.578	0.115	0.5	−1.458	0.145
Skills	T0 vs. T1	17	6.745	0.000 *	−1	1.137	0.255
	T1 vs. T2	0	0.853	0.394	0	0.577	0.564

Mdn Diff = Median difference, T0—Baseline, T1—Immediate post-intervention, T2—three-month follow-up, * statistically significant improvement if *p*-value < 0.05.

**Table 5:** Comparison of median water safety knowledge, attitudes and survival swimming scores between groups (N = 116).

Outcome	Time Points	Intervention Group (*n* = 60)	Control Group (*n* = 56)	Between Group Comparison Intervention vs. Control		
		Mdn (IQR)	Mdn (IQR)	U	Z	*p*
Knowledge	T0	6 (5–7)	6 (5–7)	1679	−0.006	0.996
	T1	13 (12–14)	7 (5–8)	3	−9.320	0.000 *
	T2	13 (12–14)	6 (5–8)	4	−9.328	0.000 *
Attitudes	T0	67 (61.25–70)	66 (62–70)	1555	−0.689	0.491
	T1	80.5 (79–82)	69.5 (66.25–74)	209	−8.140	0.000 *
	T2	80 (78–82.75)	70.0 (64.25–74)	252	−7.899	0.000 *
Skills	T0	1 (0–2)	1 (0–2.75)	1601	−0.451	0.652
	T1	18 (16–20.75)	1 (0–3)	0.0	−9.323	0.000 *
	T2	17 (16–20)	1 (0–3)	−5	9.323	0.000 *

Mdn = Median, IQR = Inter Quartile Ranges, T0—Baseline, T1—Immediate post-intervention, T2—three-month follow-up, * statistically significant improvement (*p*-value ≤ 0.05).

## Data Availability

Data may be made available upon reasonable request. Please contact corresponding author for further information.

## References

[1] van Beeck E.F., Branche C.M., Szpilman D., Modell J.H., Bierens J.J.L.M. (2005). A New Definition of Drowning: Towards Documentation and Prevention of a Global Public Health Problem. Bull. World Health Organ..

[2] World Health Organization Drowning. http://www.who.int/en/news-room/fact-sheets/detail/drowning.

[3] Peden A.E., Franklin R.C., Mahony A.J., Scarr J., Barnsley P.D. (2017). Using a retrospective cross-sectional study to analyse unintentional fatal drowning in Australia: ICD-10 coding-based methodologies verses actual deaths. BMJ Open.

[4] Tyler M.D., Richards D.B., Reske-Nielsen C., Saghafi O., Morse E.A., Carey R., Jacquet G.A. (2017). The epidemiology of drowning in low- and middle-income countries: A systematic review. BMC Public Health.

[5] He S., Lunnen J.C., Zia N., Khan U.R., Shamim K., Hyder A.A. (2015). Pattern of Presenting Complaints Recorded as Near-Drowning Events in Emergency Departments: A National Surveillance Study from Pakistan. BMC Emerg. Med..

[6] Sminkey L. (2008). World Report on Child Injury Prevention. Inj. Prev..

[7] Leavy J.E., Crawford G., Leaversuch F., Nimmo L., McCausland K., Jancey J. (2016). A Review of Drowning Prevention Interventions for Children and Young People in High, Low and Middle Income Countries. J. Community Health.

[8] Wallis B.A., Watt K., Franklin R.C., Taylor M., Nixon J.W., Kimble R.M. (2015). Interventions Associated with Drowning Prevention in Children and Adolescents: Systematic Literature Review. Inj. Prev..

[9] Sri Lanka Lifesaving (2020). Drowning Prevention Report Sri Lanka.

[10] Howland J., Mangione T., Hingson R., Smith G., Bell N., Watson R.R. (1995). Alcohol as a Risk Factor for Drowning and Other Aquatic Injuries. Alcohol, Cocaine, and Accidents.

[11] Ahlm K., Saveman B.I., Björnstig U. (2013). Drowning Deaths in Sweden with Emphasis on the Presence of Alcohol and Drugs—A Retrospective Study, 1992–2009. BMC Public Health.

[12] Croft J.L., Button C. (2015). Interacting Factors Associated with Adult Male Drowning in New Zealand. PLoS ONE.

[13] Johnson S.B., Jones V.C. (2011). Adolescent Development and Risk of Injury: Using Developmental Science to Improve Interventions. Inj. Prev..

[14] Willcox-Pidgeon S.M., Franklin R.C., Franklin R.C., Leggat P.A., Devine S. (2020). Identifying a Gap in Drowning Prevention: High-Risk Populations. Inj. Prev..

[15] Moran K. Water Safety Knowledge, Attitudes and Behaviours of Asian Youth in New Zealand. Prevention, Protection and Promotion. Proceedings of the Second International Asian Health and Wellbeing Conference.

[16] Petrass L.A., Blitvich J.D. (2014). Preventing Adolescent Drowning: Understanding Water Safety Knowledge, Attitudes and Swimming Ability. the Effect of a Short Water Safety Intervention. Accid. Anal. Prev..

[17] Moran K., Stallman R.K., Kjendlie P.L., Dahl D., Blitvich J.D., Petrass L.A., McElroy G.K., Goya T., Teramoto K., Matsui A. (2012). Can You Swim? An Exploration of Measuring Real and Perceived Water Competency. Int. J. Aquat. Res. Educ..

[18] Ministry of Disaster Management (2017). Towards a National Plan Drowning Prevention and Water Safety Sri Lanka. Colombo 05. Sri Lanka..

[19] Ekanayaka E.M.J.S.K., Rhiannon B., Mathews B., Nanayakkara A., Jayawardena M., Wijayaratne S., Dharmaratne S.D. (2018). PW 2825 Swim for Safety Sri Lanka—Survival Swimming and Water Safety Education among School Children. J. Inj. Prev..

[20] Linnan M., Rahman A., Scarr J., Reinten-Reynolds T., Linnan H., Rui-Wei J., Mashreky S., Shafinaz S., Bose S., Finkelstein E. (2012). Child Drowning: Evidence for a Newly Recognized Cause of Child Mortality in Low and Middle Income Countries in Asia.

[21] Birch R., Matthews B. (2013). Sink or Swim: The State of Victorian Primary School Children’s Swimming Ability.

[22] Petrass L.A., Simpson K., Blitvich J., Birch R., Matthews B. (2021). Exploring the Impact of a Student-Centred Survival Swimming Programme for Primary School Students in Australia: The Perceptions of Parents, Children and Teachers. Eur. Phys. Educ. Rev..

[23] Peden A., Byers B., Scarr J., Sharma P., Larsen P., Rahman A. (2017). *Survival Swimming Programs for Children: Case Studies from Canada, India**, Australia and Bangladesh*; ILS Drowning Prevention Commission; International Lifesaving Federation. https://library.ilsf.org/drowning-prevention/library/survival-swimming-programs-children-case-studies-canada-india-australia.

[24] Petrass L.A., Blitvich J.D., McElroy G.K., Harvey J., Moran K. (2012). Can You Swim? Self-Report and Actual Swimming Competence among Young Adults in Ballarat, Australia. Int. J. Aquat. Res. Educ..

[25] Golob M.I., Giles A.R., Rich K.A. (2013). Enhancing the Relevance and Effectiveness of Water Safety Education for Ethnic and Racial Minorities. Int. J. Aquat. Res. Educ..

[26] Noordzij M., Tripepi G., Dekker F.W., Zoccali C., Tanck M.W., Jager K.J. (2010). Sample Size Calculations: Basic Principles and Common Pitfalls. Nephrol. Dial. Transplant..

[27] Birch R., Matthews B., Petrass L., Blitvich J. (2015). A Pilot Study Evaluating a Before School Survival Swimming Program.

[28] Ekanayaka J., Nanayakkara A., Birch R., Matthews B., Dharmaratne S.D., Jayawardena M., Tesone L. Adapting a survival swimming programme: Challenges and solutions for successful delivery of “Swim for Safety” in Sri Lanka. Proceedings of the World Conference on Drowning Prevention.

[29] Life Saving Victoria and YMCA-Victoria (2016). Swim for Safety Programme Curriculum, Version 3.0.

[30] World Health Organization (2014). Global Report on Drowning: Preventing a Leading Killer.

[31] Life Saving Association of Sri Lanka (2014). Drowning Prevention Report.

[32] Royal Life Saving Society Commonwealth (2016). Survival Swimming Guide: Survival Swimming in Every Commonwealth Nation.

[33] Peden A., Scarr J., Bradley R., George P., Griffiths M., Larsen P., Herde E., Mallet G., Symington C., Alexander K. (2012). Australian Water Safety Strategy 2012–2015.

[34] International Life Saving Federation (2008). Drowning Prevention Strategies: A Framework to Reduce Drowning Deaths in the Aquatic Environment for Nations/Regions Engaged in Lifesaving.

[35] Stallman R.K., Moran K., Quan L., Langendorfer S. (2017). From Swimming Skill to Water Competence: Towards a More Inclusive Drowning Prevention Future. Int. J. Aquat. Res. Educ..

[36] Keogh-Brown M.R., Bachmann M.O., Shepstone L., Hewitt C., Howe A., Ramsay C.R., Song F., Miles J.N.V., Torgerson D.J., Miles S. (2007). Contamination in Trials of Educational Interventions. Health Technol. Assess..

[37] Torgerson D.J. (2001). Contamination in Trials: Is Cluster Randomisation the Answer?. BMJ.

[38] Milutinović D. (2013). Assessing Clinical Skill Competence of Nursing Students through Objective Structured Clinical Examination. SEEHSJ.

[39] Schleicher I., Leitner K., Juenger J., Moeltner A., Ruesseler M., Bender B., Sterz J., Schuettler K.F., Koenig S., Kreuder J.G. (2017). Examiner Effect on the Objective Structured Clinical Exam—A Study at Five Medical Schools. BMC Med. Educ..

[40] Royal Life Saving-Australia (2010). Water Safety for All Australians.

[41] van Griethuijsen R.A.L.F., van Eijck M.W., Haste H., den Brok P.J., Skinner N.C., Mansour N., Gencer A.S., Bou-jaoude S. (2014). Global Patterns in Students’ Views of Science and Interest in Science. Res. Sci. Educ..

[42] Seid M.A., Hussen M.S. (2018). Knowledge and Attitude towards Antimicrobial Resistance among Final Year Undergraduate Paramedical Students at University of Gondar, Ethiopia. BMC Infect. Dis..

[43] Moran K. (2008). Will They Sink or Swim? New Zealand Youth Water Safety Knowledge and Skills. Int. J. Aquat. Res. Educ..

[44] Akobeng A.K. (2005). Principles of Evidence Based Medicine. Arch. Dis. Child..

[45] Solomon R., Giganti M.J., Weiner A., Akpinar-Elci M. (2013). Water Safety Education among Primary School Children in Grenada. Int. J. Inj. Control Saf. Promot..

[46] Terzidis A., Koutroumpa A., Skalkidis I., Matzavakis I., Malliori M., Frangakis C.E., DiScala C., Petridou E.T.H. (2007). Water Safety: Age-Specific Changes in Knowledge and Attitudes Following a School-Based Intervention. Inj. Prev..

[47] Arthur W., Bennett W., Stanush P.L., McNelly T.L. (1998). Factors That Influence Skill Decay and Retention: A Quantitative Review and Analysis. Hum. Perform..

[48] Kjendlie P.L., Pedersen T., Thoresen T., Setlo T., Moran K., Stallman R.K. (2013). Can You Swim in Waves? Children’s Swimming, Floating, and Entry Skills in Calm and Simulated Unsteady Water Conditions. Int. J. Aquat. Res. Educ..

[49] Moran K. (2015). Can You Swim in Clothes? Reflections on the Perception and Reality of the Effect of Clothing on Water Competency. Int. J. Aquat. Res. Educ..

